# Supplementary material for: Patterns of Intron Gain and Loss in Fungi
Source: PLoS Biol. 2004 Nov 30;2(12):e422. doi: 10.1371/journal.pbio.0020422 (PMC532390; doi:10.1371/journal.pbio.0020422)
Supplement: Table S1 — Also available at http://genes.mit.edu/NielsenEtAl/. (4.3 MB ZIP). [file pbio.0020422.st001.zip › NielsenEtAl/html/155.html]

AN4842.1.NCU03177.1.MG03006.1.FG09542.1


```
 CLUSTAL W (1.82) Multiple Sequence Alignments - Introns Inserted


Sequence 1: NCU03177.1	303 aa
Sequence 2: FG09542.1	282 aa
Sequence 3: MG03006.1	294 aa
Sequence 4: AN4842.1	287 aa
Alignment Length: 319 aa
Number Identitical Residues: 117 aa
Alignment Score (without introns) 6403


MG03006.1 	MSRRLITTKLPSWAALEPCLNATRQQPTQTCRRFLSSPSASSPSSRRPTTTPRRIQQQQT
NCU03177.1	-----MSHAIAPMRTFSSVLSRT---PARQCQRFIST-ATTT----RPTVPRLQAQLHQP
FG09542.1 	-----MPNSVATSKALRGLFSSL---STRQCQRCFSS-SALQPKQLRPTLPRAPMQSRQP
AN4842.1  	-------MSVPLRSLMRTLARTTR--PPLLSAQCRAQVSQNTP---RCTTPSRSQWQPFS
          	         :.    :          ..  . :  :  :   .   * * .        .

MG03006.1 	FVPRRWKSETVEELKSRNRSG0PFSWKAGVIFVMTAAGLVWYMDHEKERMHKKRIADASK
NCU03177.1	VAQRRT-YKTVEEAKSKYRSG0PFSWKAGLLFVLTGAGLLWYFEHEKQRMQRKRIADATK
FG09542.1 	ITQRRTKYKTIEQAKSRYSNG0PFSWKAGFLFVGTCGLLVWYFEFEKERMQRKRIAEAAK
AN4842.1  	TTPARPKAKTMGQLRARNATG~PFSWKAALLFVLTGAGMIIYFRVEKERLERKRIAEMSK
          	 .  * . :*: : :::  .* ******..:** * . :: *:  **:*:.:****: :*

MG03006.1 	GVGKPRIGGAFELIDQDGRPFSSDSLKGRYSL~VYFGFSHCPDICPEELDKMALMFDEVQ
NCU03177.1	GVGRPKVGGPFELIDQNGKPVTEKDLKGRYSL0VYFGFSHCPDICPEELDKMAAMFEKVE
FG09542.1 	GVGRPKVGGTFELVDQDGKPFTSEMMKGKHSL0VYFGFTRCPDICPEELDKMATMLDIVE
AN4842.1  	GVGRPKVGGPFVLKDLNGDVFTEENLKGKYSF0VYFGFTHCPDICPDELDKMAEIIDKVK
          	***:*::**.* * * :*  .:.. :**::*: *****::******:****** ::: *:

MG03006.1 	KERPG--ALAPVFVTCDPERDTPEVLKEYLAEFHPDFIGLTGTYEQIKAMCKAYRVYFST
NCU03177.1	AERPG--ALKPVFVTCDPARDTPQVLKEYLAEFHPLFIGLTGTYDQIKAMCKAYRVYFST
FG09542.1 	QKAPD--ALLPIFITCDPARDTPKALKEYLSEFHDKFVGLTGTYDQIKDLCKKYRVYFST
AN4842.1  	EANKGENIFVPVFITCDPARDTPEVLRNYLQEFHKDIIGLTGTYEQVKQVCKAYRVYFST
          	    ... : *:*:**** ****:.*::** ***  ::******:*:* :** *******

MG03006.1 	PRDVKPGQDYLVDHSIYFYLMD~PEGDFVEALGRQHSPKAGANIILDHMKEYKG~-----
NCU03177.1	PSKVEPGQDYLVDHSIYFYLMD~PEGDFVEALGRQHSPDQAAKVILDHMKDWQV1LLSGS
FG09542.1 	PQNVKPGQDYLVDHSIYFYLMD~PDGDFVEALGRQHSPQQAAALILDHVKDWN-~-----
AN4842.1  	PRDVKPGEDYLVDHSIYFYLMD1PEGDFVECIGRQDTPETATKTIMEHINDWKR~--EGK
          	* .*:**:************** *:*****.:***.:*. .:  *::*:::::    ...

MG03006.1 	QLKTA------------------
NCU03177.1	DTERWCLKVLGYASGGLGSLLSL
FG09542.1 	DKK--------------------
AN4842.1  	PLKRD------------------
          	  :
```
